# Supplementary material for: Untargeted Metabolomics Identifies Potential Hypertrophic Cardiomyopathy Biomarkers in Carriers of MYBPC3 Founder Variants
Source: Int J Mol Sci. 2023 Feb 17;24(4):4031. doi: 10.3390/ijms24044031 (PMC9961357; doi:10.3390/ijms24044031)
Supplement: Supplementary file 1 [file ijms-24-04031-s001.zip › ijms-2156007-supplementary.pdf]

Untargeted Metabolomics Identifies Potential Hypertrophic Cardiomyopathy Biomarkers in Carriers of Founder Truncating *MYBPC3* Variants

Supplementary Materials

M. Jansen et al. (2023)

Table S1. Relatedness between subjects & genetic testing

Family	Subject	Group	Age	Sex	Reason for genetic testing	Type of genetic testing
		***MYBPC3* c.2373dupG p.(Trp792*fs*)**				
F001	1	No/mild phenotype	66	M	Index patient	NGS panel
F002	2	No/mild phenotype	64	M	Family member	Variant-specific
F003	3	Severe	53	M	Index patient	Sanger sequencing (*MYBPC3*)
F004	4	Severe	75	F	Index patient	NGS panel
F005	5	Severe	72	M	Index patient	NGS panel
F006	6	No/mild phenotype	63	F	Family member	Variant-specific
F007	7	Genotype-negative	52	F	Family member	Variant-specific
F008	8	Severe	20	M	Family member	Variant-specific
F009	9	Severe	49	M	Index patient	NGS panel
F010	10	Severe	44	F	Index patient	Sanger sequencing (*MYBPC3*)
	11	Severe	73	M	Family member	Variant-specific
F011	12	No/mild phenotype	63	F	Family member	Variant-specific
	13	No/mild phenotype	70	F	Family member	Variant-specific
F012	14	No/mild phenotype	70	M	Family member	Variant-specific
	15	No/mild phenotype	75	M	Family member	Variant-specific
F013	16	Genotype-negative	66	M	Family member	Variant-specific
F014	17	Genotype-negative	23	M	Family member	Variant-specific
F015	18	Severe	41	M	Index patient	NGS panel
	19	Severe	37	M	Family member	Variant-specific
F016	20	Severe	67	M	Index patient	Sanger sequencing (*MYBPC3*)
F017	21	No/mild phenotype	38	M	Family member	Variant-specific
	22	Genotype-negative	60	F	Family member	Variant-specific
F018	23	Severe	58	M	Index patient	Sanger sequencing (*MYBPC3*)
F019	24	Genotype-negative	67	F	Family member	Variant-specific
	25	Genotype-negative	46	M	Family member	Variant-specific
F020	26	No/mild phenotype	57	M	Family member	Variant-specific
F021	27	Severe	73	F	Family member	Variant-specific
F022	28	No/mild phenotype	30	F	Family member	Variant-specific
F023	29	Severe	26	F	Index patient	NGS panel
F024	30	No/mild phenotype	72	F	Family member	Variant-specific
	31	No/mild phenotype	48	F	Family member	Variant-specific
F025	32	No/mild phenotype	57	M	Family member	Variant-specific
F026	33	No/mild phenotype	23	M	Family member	Variant-specific
F027	34	Severe	68	M	Index patient	NGS panel
F028	35	Severe	55	M	Index patient	NGS panel
F029	36	Genotype-negative	59	M	Family member	Variant-specific

Family	Subject	Group	Age	Sex	Reason for genetic testing	Type of genetic testing
F030	37	Severe	72	F	Family member	Variant-specific
	38	Severe	69	M	Family member	Variant-specific
F031	39	No/mild phenotype	28	M	Index patient	NGS panel
F032	40	No/mild phenotype	24	F	Family member	Variant-specific
	41	No/mild phenotype	26	M	Family member	Variant-specific
	42	No/mild phenotype	54	F	Family member	Variant-specific
F033	43	Genotype-negative	40	M	Family member	Variant-specific
F034	44	No/mild phenotype	69	M	Family member	Variant-specific
F035	45	No/mild phenotype	67	F	Family member	Variant-specific
F036	46	No/mild phenotype	72	M	Index patient	Sanger sequencing (*MYBPC3*, *MYH7*)
	47	No/mild phenotype	39	M	Family member	Variant-specific
F037	48	Severe	54	F	Index patient	Sanger sequencing (*MYBPC3*)
F038	49	Severe	77	M	Index patient	Variant-specific
F039	50	No/mild phenotype	60	F	Family member	Variant-specific
F040	51	No/mild phenotype	38	F	Family member	Variant-specific
	52	No/mild phenotype	52	M	Family member	Variant-specific
F041	53	Genotype-negative	72	M	Family member	Variant-specific
F042	54	Severe	71	F	Index patient	Variant-specific
	55	Severe	68	F	Family member	Variant-specific
	56	No/mild phenotype	31	M	Family member	Variant-specific
	57	Severe	36	M	Family member	Variant-specific
	58	Severe	39	M	Family member	Variant-specific
	59	No/mild phenotype	42	M	Family member	Variant-specific
		***MYBPC3* c.2827C>T p.(Arg943*)**				
F043	60	Severe	27	M	Index patient	NGS panel
F044	61	Severe	29	F	Index patient	NGS panel
F045	62	No/mild phenotype	79	M	Index patient	Sanger sequencing (*MYBPC3*, *MYH7*)
	63	No/mild phenotype	70	F	Family member	Variant-specific
		***MYBPC3* c.2864_2865delCT p.(Pro955*fs*)**				
F046	64	Severe	73	F	Index patient	NGS panel
	65	Severe	73	F	Family member	Variant-specific
F047	66	Severe	27	M	Index patient	NGS panel
F048	67	Severe	61	F	Family member	Variant-specific
F049	68	Genotype-negative	40	F	Family member	Variant-specific
F050	69	No/mild phenotype	66	F	Index patient	NGS panel
		***MYBPC3* c.3776delA p.(Gln1259*fs*)**				

Family	Subject	Group	Age	Sex	Reason for genetic testing	Type of genetic testing
F051	70	Severe	38	F	Family member	Variant-specific

Data shown as counts (%). F, female; M, male; NGS, Next-Generation Sequencing.

Table S2. Peaks included in biomarker identification models

Overall rank	Metabolite name(s)	sPLS-DA weight	sPLS-DA rank	XGBoost gain	XGBoost rank	Lasso coefficient	Lasso rank
1	[Threoninyl-Tryptophan, Tryptophyl-Threonine]	3.43E-01	2	3.72E-02	4	-5.59E-01	2
2	[2-Methoxy-estradiol-17b 3-glucuronide, 4-hydroxyandrostenedione glucuronide]	3.44E-01	1	6.27E-02	1	-2.22E-01	6
3	Menadiol disuccinate	-2.24E-01	8			7.12E-01	1
4	Aminoadipic acid	-2.32E-01	6	3.58E-02	5	2.88E-01	5
5	[Aspartyl-(iso)leucine, L-beta-aspartyl-L-leucine, (gamma-)glutamylvaline, (iso)leucyl-aspartate]	-2.71E-01	3	4.58E-03	60	2.97E-01	4
6	[Norophthalmic acid, N2-gamma-Glutamylglutamine]	2.51E-01	4	1.66E-02	15	-1.27E-01	9
7	[1,3-Dimethyluracil, Imidazolepropionic acid, (pi-)Methylimidazoleacetic acid]	-2.09E-01	9	2.68E-02	7		
8	[cis-Zeatin O-glucoside, cis-Zeatin-7/9-N-glucoside]	1.66E-01	12			-3.12E-01	3
9	3-Fumarylpyruvate	1.85E-01	10			-2.13E-01	7
10	9,12-Hexadecadienoylcarnitine			4.44E-02	2		
11	2-Octenoylcarnitine	-1.47E-01	17			1.94E-01	8
12	Ceramide(t18:0/16:0)	2.40E-01	5			-8.67E-07	11
13	[(Iso)nicotinic acid, picolinic acid]	-1.26E-01	19	1.87E-02	12		
14	4-Trimethylammoniobutanal	-2.25E-01	7			1.82E-06	10
15	[(S)-3,4-/2,4-Dihydroxybutyric acid, 4-deoxythreonic/-erythronic acid, erythrose, L-erythrulose]	-1.62E-01	13	1.11E-02	28		
16	3-Oxoalanine	-8.68E-03	45	3.84E-02	3		
17	Uric acid	-1.16E-01	22	1.58E-02	17		
18	[Hydroxyprolyl-Histidine, Histidylhydroxyproline]	-1.18E-01	21	1.30E-02	22		

Overall rank	Metabolite name(s)	sPLS-DA weight	sPLS-DA rank	XGBoost gain	XGBoost rank	Lasso coefficient	Lasso rank
19	[Dextrin, D-Gal alpha 1->6D-Gal alpha 1->6D-glucose, 3-galactosyllactose, 1-kestose, maltotriose, melezitose]	-1.71E-01	11	2.11E-03	97		
20	3,4-Dihydroxybenzylamine			2.93E-02	6		
21	Hydantoin-5-propionic acid	-1.49E-01	16	1.77E-03	103		
22	Vanilloylglycine	1.56E-01	14				
23	6-Carboxy-5,6,7,8-tetrahydropterin	-1.53E-01	15				
24	Methylguanidine	-1.40E-01	18				
25	[all-trans-Hexaprenyl/Presqualene diphosphate]	-1.05E-01	25	6.05E-03	46		
26	Mesobilirubinogen			2.21E-02	8		
27	Protoporphyrin IX	8.02E-02	28	7.17E-03	38		
28	[Deoxyhypusine, Spermic acid 1]	-1.18E-01	20				
29	Arachidyl carnitine			2.07E-02	9		
30	Ethyl 3-furoate	-8.58E-02	27	5.05E-03	52		
31	[Serinyl-Threonine, Threonylserine]			2.06E-02	10		
32	2-(3-Carboxy-3-(methylammonio)propyl)-L-histidine	-1.12E-01	23				
33	3-Methylhistidine	-1.10E-01	24				
34	[5,6-/ 8,9-/11,12-/14,15-Dihydroxyeicosatrienoic acid, 6,7-dihydro-12-epi-/10,11-dihydro-/12-keto-tetrahydro-leukotriene B4, 15-hydroperoxyeicosa-8Z,11Z,13E-trienoate]			1.98E-02	11		
35	[Tryptophyl-Tyrosine, Tyrosyl-Tryptophan]			1.85E-02	13		
36	Guanidinosuccinic acid	-4.57E-02	33	9.24E-03	33		
37	[Tetracosahexaenoic acid(, n-3)]			1.69E-02	14		
38	6,7-Dimethyl-8-(1-D-ribityl)lumazine	-8.89E-02	26				
39	Serinyl-Serine	-5.95E-02	31	5.01E-03	54		

Overall rank	Metabolite name(s)	sPLS-DA weight	sPLS-DA rank	XGBoost gain	XGBoost rank	Lasso coefficient	Lasso rank
40	[Cholestane-3b,5a,6b-triol, 5b-cholestane-3a,7a,12a-triol/-3a,7a,26-triol/-3a,7a,27-triol]			1.59E-02	16		
41	[Hexanoylglycine, isovalerylalanine/-sarcosine, N-acetylleucine]			1.56E-02	18		
42	[D-/L-Lactic acid, hydroxypropionic acid, glyceraldehyde, dihydroxyacetone, methoxyacetic acid]			1.45E-02	19		
43	[D-/L-Aspartic acid, Iminodiacetic acid]			1.41E-02	20		
44	D-Glucuronic acid 1-phosphate	-6.70E-02	29	1.39E-03	113		
45	[(3-)Oxoglutaric acid]			1.35E-02	21		
46	5-Methylthioribose	-6.64E-02	30	1.07E-03	133		
47	1-Phenyl-5-propyl-1H-pyrazole			1.25E-02	23		
48	[3-Methoxy-4-hydroxyphenyl(ethylene)glycol sulfate]			1.22E-02	24		
49	Betaine aldehyde			1.21E-02	25		
50	Neuraminic acid	-5.44E-02	32	2.06E-03	99		
51	[(1R)-Glutathionyl-(2R)-hydroxy-1,2-dihydronaphthalene, (1R/1S)-Hydroxy-(2R/2S)-glutathionyl-1,2-dihydronaphthalene]			1.16E-02	26		
52	3-Hydroxyanthranilic acid			1.12E-02	27		
53	albendazole S-oxide			1.10E-02	29		
54	[24-Hydroxycalcitriol, 3beta,7alpha-Dihydroxy-5-cholestenoate, 23S,25,26-Trihydroxyvitamin D3]	-3.42E-02	38	4.24E-03	64		
55	Lysosphingomyelin(d18:1)	-3.76E-02	36	3.47E-03	77		
56	Stearidonyl carnitine	-2.80E-03	48	9.73E-03	30		
57	[Glutamyl(-Iso)leucine, (Iso)leucyl-Glutamate]			9.65E-03	31		
58	Deoxyuridine-diphosphate			9.57E-03	32		
59	[MG(0:0/22:0/0:0), MG(22:0/0:0/0:0)]	-1.67E-02	43	6.08E-03	45		

Overall rank	Metabolite name(s)	sPLS-DA weight	sPLS-DA rank	XGBoost gain	XGBoost rank	Lasso coefficient	Lasso rank
60	Histidylproline diketopiperazine			8.88E-03	34		
61	[Asparaginyl-Cysteine, Cysteinyl-Asparagine]			8.44E-03	35		
62	Indanone	-2.66E-02	40	3.48E-03	76		
63	[MG(0:0/20:2(11Z,14Z)/0:0), MG(20:2(11Z,14Z)/0:0/0:0)]	-4.41E-02	34				
64	[D-/L-/DL-Glutamic acid, N-Methyl-D-aspartic acid, N-Acetylserine, L-4-Hydroxyglutamate semialdehyde]	-7.22E-03	46	6.63E-03	41		
65	Cis-stilbene oxide	-4.22E-02	35				
66	[Sinapyl alcohol, 3,4-Dihydroxyphenylvaleric acid]			7.61E-03	36		
67	E,e-Carotene-3,3'-dione			7.57E-03	37		
68	[cis-2-Methylaconitate, (Z)-But-1-ene-1,2,4-tricarboxylate]			7.06E-03	39		
69	[Homoanserine, Histidylvaline, Valylhistidine]			6.79E-03	40		
70	1-Lyso-2-arachidonoyl-phosphatidate	3.65E-02	37				
71	3-Hydroxyquinine			6.60E-03	42		
72	24,25,26,27-Tetranor-23-oxo-hydroxyvitamin D3			6.41E-03	43		
73	Psychosine sulfate			6.14E-03	44		
74	[N-Acetyl-6-O-L-fucosyl-D-glucosamine, 2-Acetamido-2-deoxy-6-O-a-L-fucopyranosyl-D-glucose, 3-O-fucopyranosyl-2-acetamido-2-deoxyglucopyranose]			5.94E-03	47		
75	[Alanyl-Methionine, Cysteinyl-Valine, Methionyl-Alanine, Valylcysteine]			5.94E-03	48		
76	[Indoleacetic acid, 5-Hydroxyindoleacetaldehyde]	-3.15E-02	39				
77	Pyrocatechol sulfate			5.74E-03	49		
78	Quercetin 3'-O-glucuronide			5.66E-03	50		

Overall rank	Metabolite name(s)	sPLS-DA weight	sPLS-DA rank	XGBoost gain	XGBoost rank	Lasso coefficient	Lasso rank
79	Indole-5,6-quinone	-1.94E-02	42	2.02E-03	100		
80	[Glutaminyl-Tyrosine, Tyrosyl-Glutamine, Tyrosyl-Gamma-glutamate, gamma-Glutamyltyrosine]			5.15E-03	51		
81	[(alpha-/beta-)D-Glucose, D-/L-Galactose, D-Mannose, myo-/scyllo-Inositol, 3-Deoxyarabinohexonic acid, D-Fructose, Allose, L-Sorbose, D-Tagatose, L-Gulose]			5.02E-03	53		
82	[(D-)4-Hydroxy-2-oxoglutaric acid]			4.96E-03	55		
83	Pyrrole-2-carboxylic acid			4.89E-03	56		
84	Glycine			4.88E-03	57		
85	2-Hydroxypyridine			4.78E-03	58		
86	[N-Acetyl-D-Glucosamine 6-Phosphate, N-Acetyl-D-mannosamine 6-phosphate, N-Acetyl-glucosamine 1-/6-phosphate, N-Acetyl-(alpha-)D-galactosamine 1-phosphate]			4.69E-03	59		
87	Isoxanthopterin			4.52E-03	61		
88	Deoxycytidine-triphosphate			4.47E-03	62		
89	[Asparaginyl-Glycine, Glycyl-Asparagine, Glycylglycylglycine]	-2.45E-02	41				
90	2H3-Glutamate (internal standard)			4.36E-03	63		
91	4-Oxo-2-nonenal			4.19E-03	65		
92	Arsenate			4.08E-03	66		
93	Putrescine			4.07E-03	67		
94	N-[(4E,8E)-1,3-dihydroxyoctadeca-4,8-dien-2-yl]hexadecanamide			4.07E-03	68		
95	[L-/DL-Dopa, N-Hydroxy-L-tyrosine]			4.03E-03	69		
96	Flavone			3.93E-03	70		
97	2-Phenylethanol glucuronide			3.88E-03	71		

Overall rank	Metabolite name(s)	sPLS-DA weight	sPLS-DA rank	XGBoost gain	XGBoost rank	Lasso coefficient	Lasso rank
98	[2-Chloro-5-methylmaleylacetate, 3-Chloro-2-methylmaleylacetate]	-1.45E-02	44	1.07E-03	134		
99	Indolepyruvate			3.69E-03	72		
100	[4-Hydroxybenzoic acid, Gentisate aldehyde]			3.68E-03	73		
101	Topaquinone			3.66E-03	74		
102	Adenylylselenate			3.57E-03	75		
103	[MG(0:0/15:0/0:0), MG(15:0/0:0/0:0)]			3.46E-03	78		
104	Hydroxyprolyl-Hydroxyproline			3.41E-03	79		
105	S-Nitrosoglutathione			3.31E-03	80		
106	Indoxyl sulfate	-4.89E-03	47	2.40E-03	90		
107	2-Hydroxyundecanoate			3.26E-03	81		
108	3-Hydroxydodecanedioic acid			3.17E-03	82		
109	L-Glutamic acid 5-phosphate			3.12E-03	83		
110	2'-Deoxyinosine triphosphate			3.09E-03	84		
111	Neuromedin N (1-4)			2.97E-03	85		
112	[2-Phenylglycine, Dopamine quinone, Leukoaminochrome]			2.54E-03	86		
113	Bisnorbiotin			2.54E-03	87		
114	[7b-Hydroxy-3-oxo-5b-cholanoic acid, MG(0:0/22:6(4Z,7Z,10Z,13Z,16Z,19Z)/0:0), MG(22:6(4Z,7Z,10Z,13Z,16Z,19Z)/0:0/0:0)]			2.54E-03	88		
115	8-Oxoguanine			2.42E-03	89		
116	[Lysyl-Threonine, Threonyllysine]			2.38E-03	91		
117	Galactosylglycerol			2.32E-03	92		
118	[DG(14:0/14:0/0:0), DG(14:0/0:0/14:0)]			2.31E-03	93		
119	[Arginyl-(gamma-)Glutamine, (gamma-)Glutaminylarginine]			2.26E-03	94		
120	[Glucosamine 6-sulfate, N-Sulfo-D-glucosamine]			2.23E-03	95		

Overall rank	Metabolite name(s)	sPLS-DA weight	sPLS-DA rank	XGBoost gain	XGBoost rank	Lasso coefficient	Lasso rank
121	L-Palmitoylcarnitine			2.16E-03	96		
122	Ciliatine			2.08E-03	98		
123	Pentosidine			1.90E-03	101		
124	Xanthurenic acid			1.84E-03	102		
125	Lysosphingomyelin(d18:0)			1.74E-03	104		
126	Menadione			1.71E-03	105		
127	[(Iso)peonidin-3-glucoside, Isopeonidin 3-galactoside]			1.69E-03	106		
128	Dimethylprotoporphyrin IX dimethyl ester			1.56E-03	107		
129	[Uridine diphosphate glucose/galactose]			1.54E-03	108		
130	[Xanthine, 6,8-Dihydroxypurine]			1.49E-03	109		
131	[Lysophosphatidic acid(0:0/18:0), Lysophosphatidic acid(18:0/0:0)]			1.47E-03	110		
132	Diethylthiophosphate			1.41E-03	111		
133	[S-(Hydroxymethyl)glutathione, gamma-Glutamylcysteinylserine]			1.40E-03	112		
134	[Guanosine triphosphate, 8-Oxo-Deoxyguanosine triphosphate]			1.36E-03	114		
135	Dihydroneopterin triphosphate			1.35E-03	115		
136	Sedoheptulose 1,7-bisphosphate			1.34E-03	116		
137	[4-Hydroxyphenylpyruvic acid, 2-Hydroxy-3-(4-hydroxyphenyl)propenoic acid, 3-Hydroxyphenylpyruvic acid\			1.32E-03	117		
138	[Alanyl-Tyrosine, Phenylalanylserine, Serylphenylalanine, Tyrosyl-Alanine]			1.30E-03	118		
139	4-Hydroperoxy-2-nonenal			1.29E-03	119		

Overall rank	Metabolite name(s)	sPLS-DA weight	sPLS-DA rank	XGBoost gain	XGBoost rank	Lasso coefficient	Lasso rank
140	[Cholesteryl acetate, 4alpha-Formyl-4beta-methyl-5alpha-cholesta-8-en-3beta-ol, 4alpha-Hydroxymethyl-4beta-methyl-5alpha-cholesta-8,24-dien-3beta-ol, 3-beta-Hydroxy-4-beta-methyl-5-alpha-cholest-7-ene-4-alpha-carbaldehyde]			1.26E-03	120		
141	[2-O-a-L-Fucopyranosyl-galactose, 3-O-a-L-Fucopyranosyl-D-glucose]			1.26E-03	121		
142	15N_2-13C-Glycine (internal standard)			1.25E-03	122		
143	Glycylprolylhydroxyproline			1.25E-03	123		
144	[Chenodeoxycholic acid disulfate, Deoxycholic acid disulfate]			1.25E-03	124		
145	gamma-Glutamyl-beta-aminopropiononitrile			1.22E-03	125		
146	[DG(14:0/20:1(11Z)/0:0), DG(14:1(9Z)/20:0/0:0), DG(16:0/18:1(11Z)/0:0), DG(16:0/18:1(9Z)/0:0), DG(16:1(9Z)/18:0/0:0), DG(18:0/16:1(9Z)/0:0), DG(18:1(11Z)/16:0/0:0), DG(18:1(9Z)/16:0/0:0), DG(20:0/14:1(9Z)/0:0), DG(20:1(11Z)/14:0/0:0), DG(14:0/0:0/20:1n9), DG(16:0/0:0/18:1n7), DG(16:0/0:0/18:1n9), DG(18:0/0:0/16:1n7), DG(20:0/0:0/14:1n5)]			1.21E-03	126		
147	Deoxycytidine monophosphate			1.20E-03	127		
148	S-(2-Methylpropionyl)-dihydrolipoamide-E			1.14E-03	128		
149	Ceramide phosphate(d18:1/12:0)			1.13E-03	129		
150	L-Cysteinylglycine disulfide			1.13E-03	130		
151	L-Cystine			1.11E-03	131		
152	[(Iso)leucyl-(Iso)leucine]			1.07E-03	132		
153	[5-Methylthiorib(ul)ose 1-phosphate]			1.06E-03	135		

Overall rank	Metabolite name(s)	sPLS-DA weight	sPLS-DA rank	XGBoost gain	XGBoost rank	Lasso coefficient	Lasso rank
154	Guanethidine			1.00E-03	136		
155	[2-(3-Carboxy-3-aminopropyl)-L-histidine, Histidylthreonine, Threonylhistidine]			9.83E-04	137		
156	S-(1,2-Dichlorovinyl)glutathione			9.83E-04	138		
157	[Gamma-glutamyl-L-putrescine, Alanyllysine, Lysylalanine, beta-Alanyl-L-lysine]			9.70E-04	139		
158	Quercetin 3'-sulfate			9.61E-04	140		
159	[D-/L-Glutamine, Ureidoisobutyric acid, Alanylglycine]			9.48E-04	141		
160	[Glycyl-Lysine, Lysylglycine]			9.40E-04	142		
161	Sumiki's acid			9.37E-04	143		
162	[3b,12a-Dihydroxy-5a-cholanoic acid, 3b,7a-Dihydroxy-5b-cholanoic acid, 3a,7a-Dihydroxycholanoic acid, 3a,12b-Dihydroxy-5b-cholanoic acid, 3b,12a-Dihydroxy-5b-cholanoic acid, (Allo/iso)deoxycholic acid, (Allo)chenodeoxycholic acid, (Iso)hyodeoxycholic acid, (Iso)ursodeoxycholic acid, Murocholic acid, 7b,12a-Dihydroxycholanoic acid, 7a,12b-dihydroxy-5b-Cholan-24-oic acid, 3beta,12beta-Dihydroxy-5beta-cholanoic acid]			9.37E-04	144		
163	O-Phospho-4-hydroxy-L-threonine			9.19E-04	145		
164	Temurin			9.12E-04	146		
165	Selenocysteine	-2.29E-03	49				
166	Saccharopine	-1.07E-03	50				

Full list of the peaks included in each of the biomarker identification models (Sparse partial least squares discriminant analysis, XGBoost and Lasso logistic regression). Coefficients and ranks are provided for each separate model, Peaks have been ordered by their overall relative importance. Names of metabolites annotated to the same peak are listed within square brackets. sPLS-DA, Sparse partial least squares discriminant analysis; MG, Monoacylglycerol; DG, diacylglycerol.

Table S3. Sensitivity analyses: Lasso logistic regression results

	Excluding heart failure<br>Odds ratio	Excluding prior septal reduction therapy<br>Odds ratio
[Threoninyl-Tryptophan, Tryptophyl-Threonine]	0.260	0.302
[2-Methoxy-estradiol-17b 3-glucuronide, 4-hydroxyandrostenedione glucuronide]	Not selected	0.791
Menadiol disuccinate	1.572	1.091
Aminoadipic acid	1.000	Not selected
[Aspartyl-(iso)leucine, L-beta-aspartyl-L-leucine, (gamma-)glutamylvaline, (iso)leucyl-aspartate]	4.101	Not selected
[Norophthalmic acid, N2-gamma-Glutamylglutamine]	0.205	0.488
[1,3-Dimethyluracil, imidazolepropionic acid, (Pi-)methylimidazoleacetic acid]	Not selected	Not selected
[cis-Zeatin O-glucoside, Cis-zeatin-7/9-N-glucoside]	0.300	0.366
3-Fumarylpyruvate	0.740	0.572
9,12-Hexadecadienoylcarnitine	0.936	0.930
2-Octenoylcarnitine	1.015	Not selected
Cer(t18:0/16:0)	0.935	0.648
[(Iso)nicotinic acid, picolinic acid]	Not selected	Not selected
4-Trimethylammoniobutanal	Not selected	1.239
[(S)-3,4-/2,4-Dihydroxybutyric acid, 4-deoxythreonic/-erythronic acid, erythrose, L-erythrulose]	1.140	Not selected
3-Oxoalanine	Not selected	1.237
Uric acid	Not selected	1.060
[Hydroxyprolyl-Histidine, Histidylhydroxyproline]	1.198	1.867
[Dextrin, D-Gal alpha 1->6D-Gal alpha 1->6D-glucose, 3-galactosyllactose, 1-kestose, maltotriose, melezitose]	1.155	Not selected
3,4-Dihydroxybenzylamine	2.732	Not selected
Hydantoin-5-propionic acid	Not selected	Not selected
Vanilloylglycine	Not selected	0.860

	Excluding heart failure Odds ratio	Excluding prior septal reduction therapy Odds ratio
6-Carboxy-5,6,7,8-tetrahydropterin	1.688	1.432
Methylguanidine	Not selected	Not selected
[all-trans-Hexaprenyl/Presqualene diphosphate]	1.295	1.719
Mesobilirubinogen	Not selected	Not selected
[Deoxyhypusine, Spermic acid 1]	Not selected	Not selected
Arachidyl carnitine	1.400	Not selected
[Serinyl-Threonine, Threonylserine]	Not selected	Not selected
2-(3-Carboxy-3-(methylammonio)propyl)-L-histidine	Not selected	Not selected
3-Methylhistidine	Not selected	Not selected
[5,6-/ 8,9-/11,12-/14,15-Dihydroxyeicosatrienoic acid, 6,7-dihydro-12-epi-/10,11-dihydro-/12-keto-tetrahydro-leukotriene B4, 15-hydroperoxyeicosa-8Z,11Z,13E-trienoate]	5.202	Not selected
[Tryptophyl-Tyrosine, Tyrosyl-Tryptophan]	0.735	0.953
[Tetracosahexaenoic acid(, n-3)]	Not selected	Not selected
[Cholestane-3b,5a,6b-triol, 5b-cholestane-3a,7a,12a-triol/-3a,7a,26-triol/-3a,7a,27-triol]	Not selected	Not selected
[Hexanoylglycine, isovalerylalanine/-sarcosine, N-acetylleucine]	Not selected	Not selected
[D-/L-Lactic acid, hydroxypropionic acid, glyceraldehyde, dihydroxyacetone, methoxyacetic acid]	Not selected	1.280
[D-/L-Aspartic acid, Iminodiacetic acid]	Not selected	Not selected
[(3-)Oxoglutaric acid]	Not selected	1.236
1-Phenyl-5-propyl-1H-pyrazole	0.973	0.901
[3-Methoxy-4-hydroxyphenyl(ethylene)glycol sulfate]	Not selected	Not selected
Betaine aldehyde	Not selected	Not selected

Results of Lasso logistic regression in both sensitivity analyses. The first excluded subjects with heart failure (congestive heart failure or left ventricular ejection fraction <50%), the second excluded subjects with prior septal reduction therapy. Metabolites are ordered by their overall rank across the metabolite selection models employed on the full cohort, as in Supplemental Table 2.

Table S4. External validation: Lasso logistic regression results

	Odds ratio
Male sex	1.396E+06
Age	2.511E+04
[Threoninyl-Tryptophan, Tryptophyl-Threonine]	4.405
[2-Methoxy-estradiol-17b 3-glucuronide, 4-hydroxyandrostenedione glucuronide]	2.062E+02
Menadiol disuccinate	5.708E+02
Aminoadipic acid	1.409
[Aspartyl-(iso)leucine, L-beta-aspartyl-L-leucine, (gamma-)glutamylvaline, (iso)leucyl-aspartate]	Not selected
[Norophthalmic acid, N2-gamma-Glutamylglutamine]	Not selected
[1,3-Dimethyluracil, imidazolepropionic acid, (Pi-)methylimidazoleacetic acid]	0.028
[cis-Zeatin O-glucoside, Cis-zeatin-7/9-N-glucoside]	Not selected
3-Fumarylpyruvate	2.416
9,12-Hexadecadienoylcarnitine	0.070

Results of Lasso logistic regression in both sensitivity analyses. The first excluded subjects with heart failure (congestive heart failure or left ventricular ejection fraction <50%), the second excluded subjects with prior septal reduction therapy. Metabolites are ordered by their overall rank across the metabolite selection models employed on the full cohort, as in Supplemental Table 2.

Figure S1. Distribution of outcomes

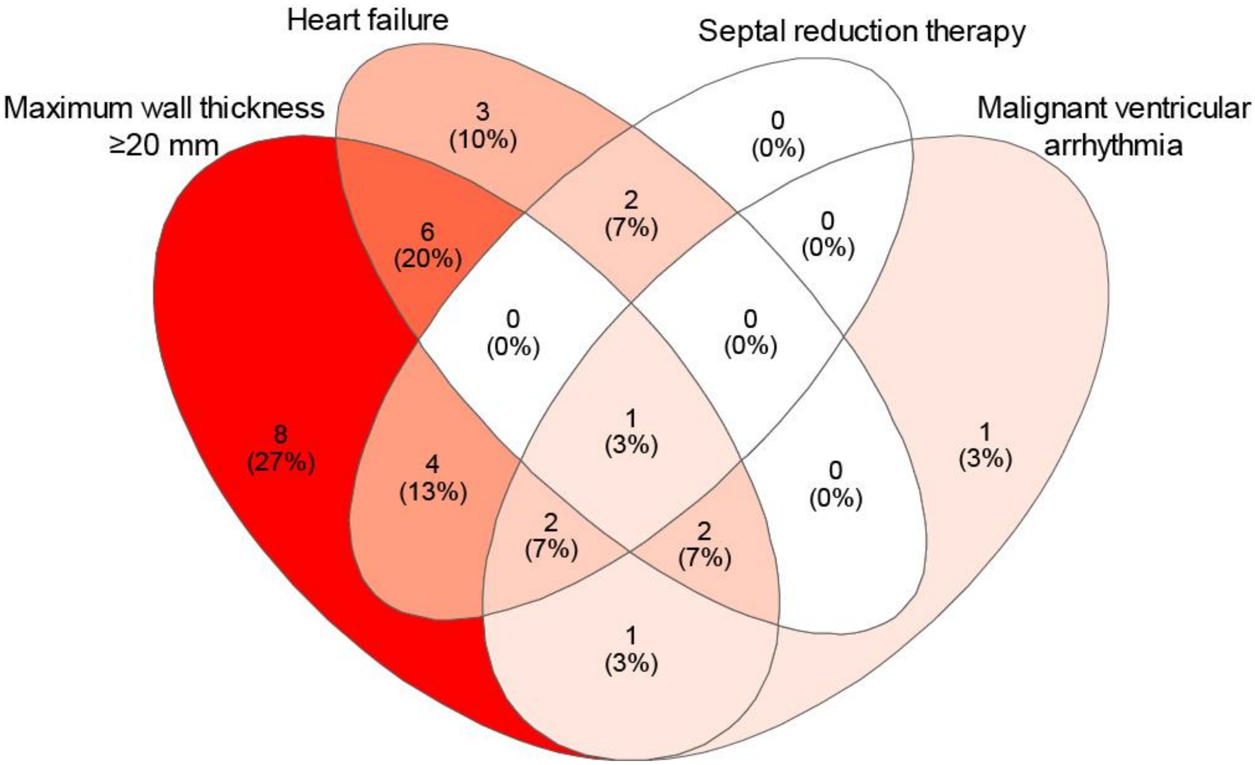

Venn diagram of outcomes fulfilled by the subjects with severe phenotypes, created using the ggVennDiagram package. The numbers in each section represent the number of patients fulfilling each combination of outcomes. Colours are scaled to the number of subjects per section.

Figure S2. Correlation between peaks

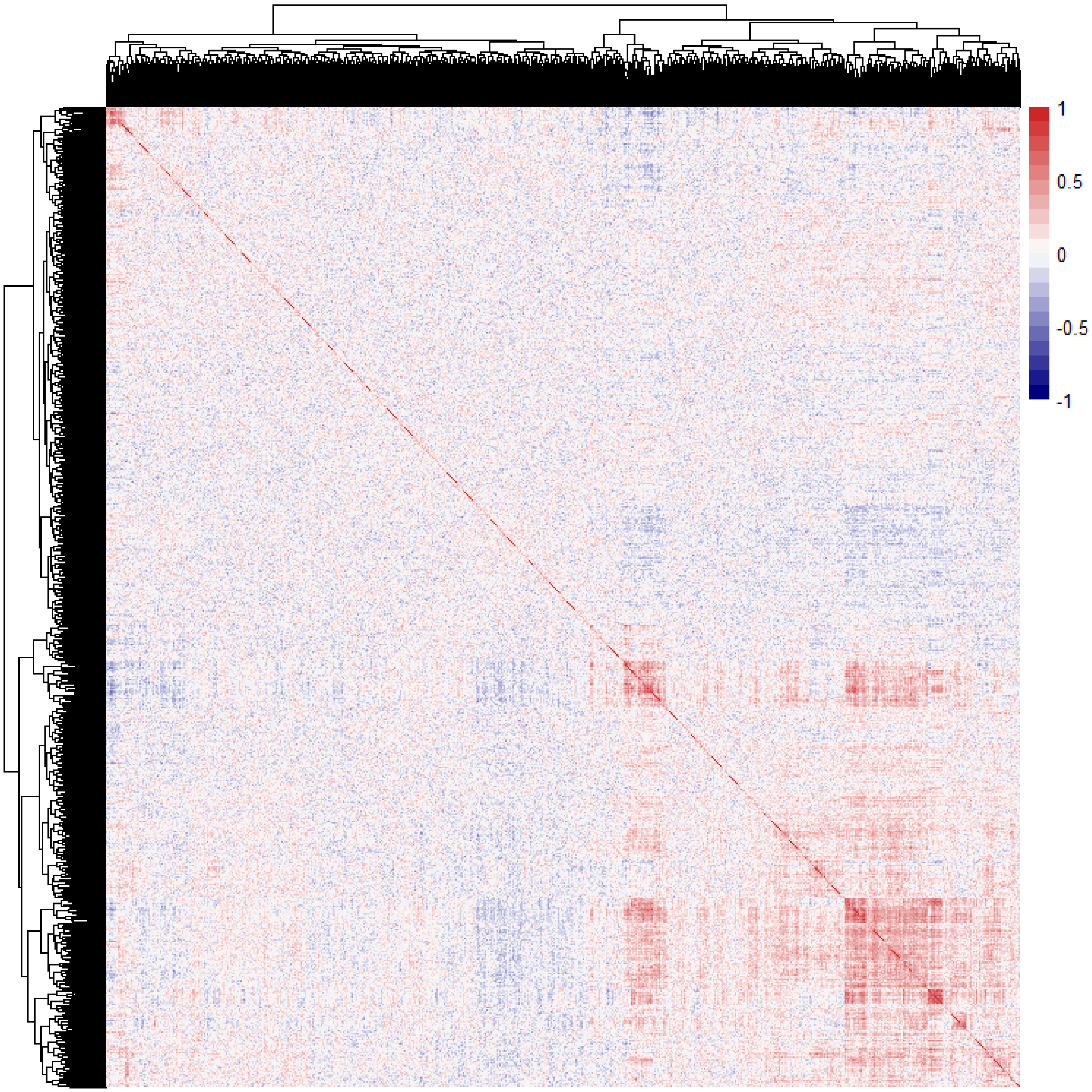

Heatmap of Spearman’s correlation coefficients between the 1903 unique metabolite peaks.

Figure S3. Correlations between the selected peaks

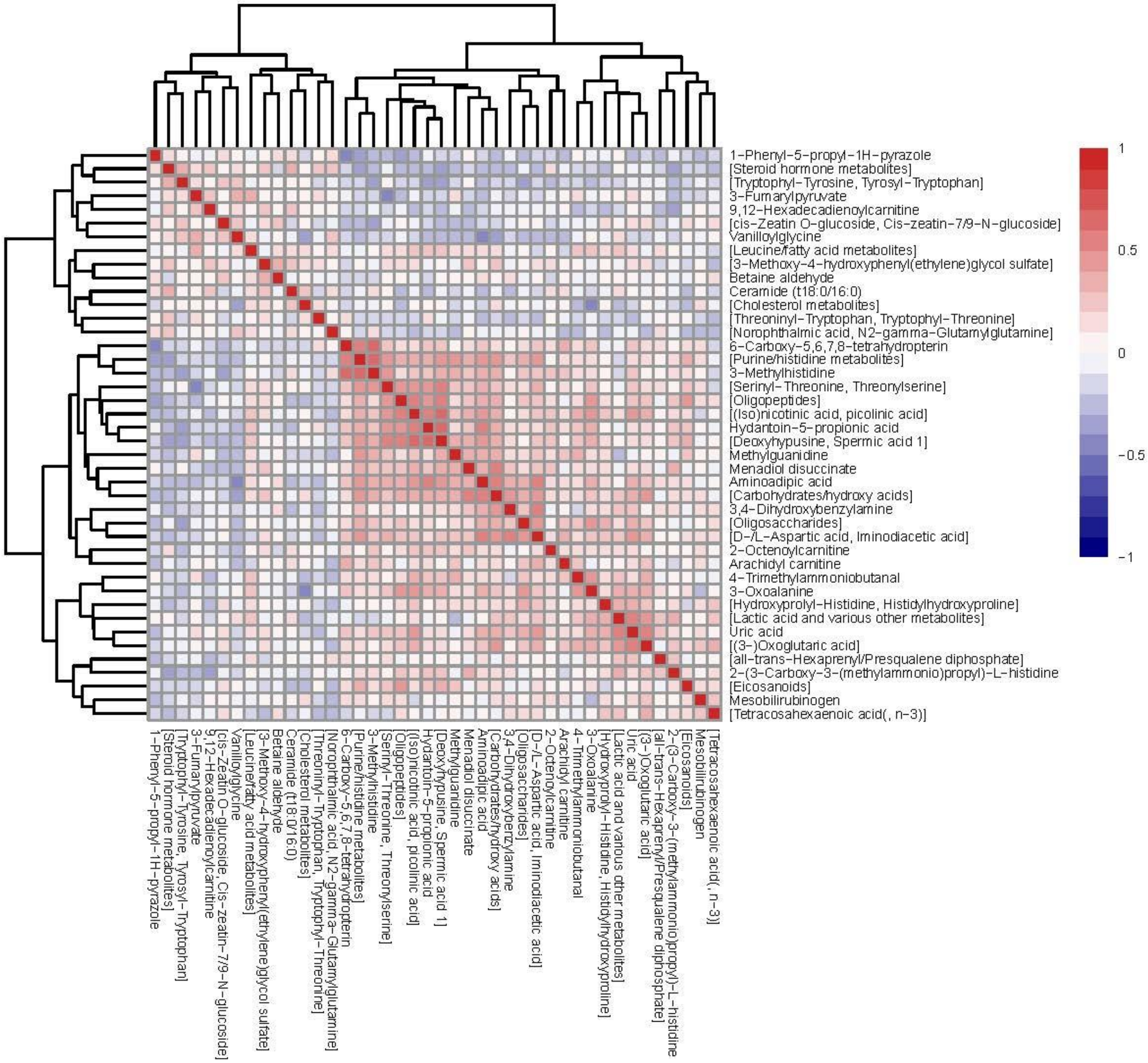

Heatmap of Spearman's ρ correlations between the 42 top metabolite peaks, consisting of the top 25 peaks identified by each analysis (sparse partial least squares discriminant analysis, XGBoost and Lasso logistic regression).

Figure S4. Box plots of selected peaks

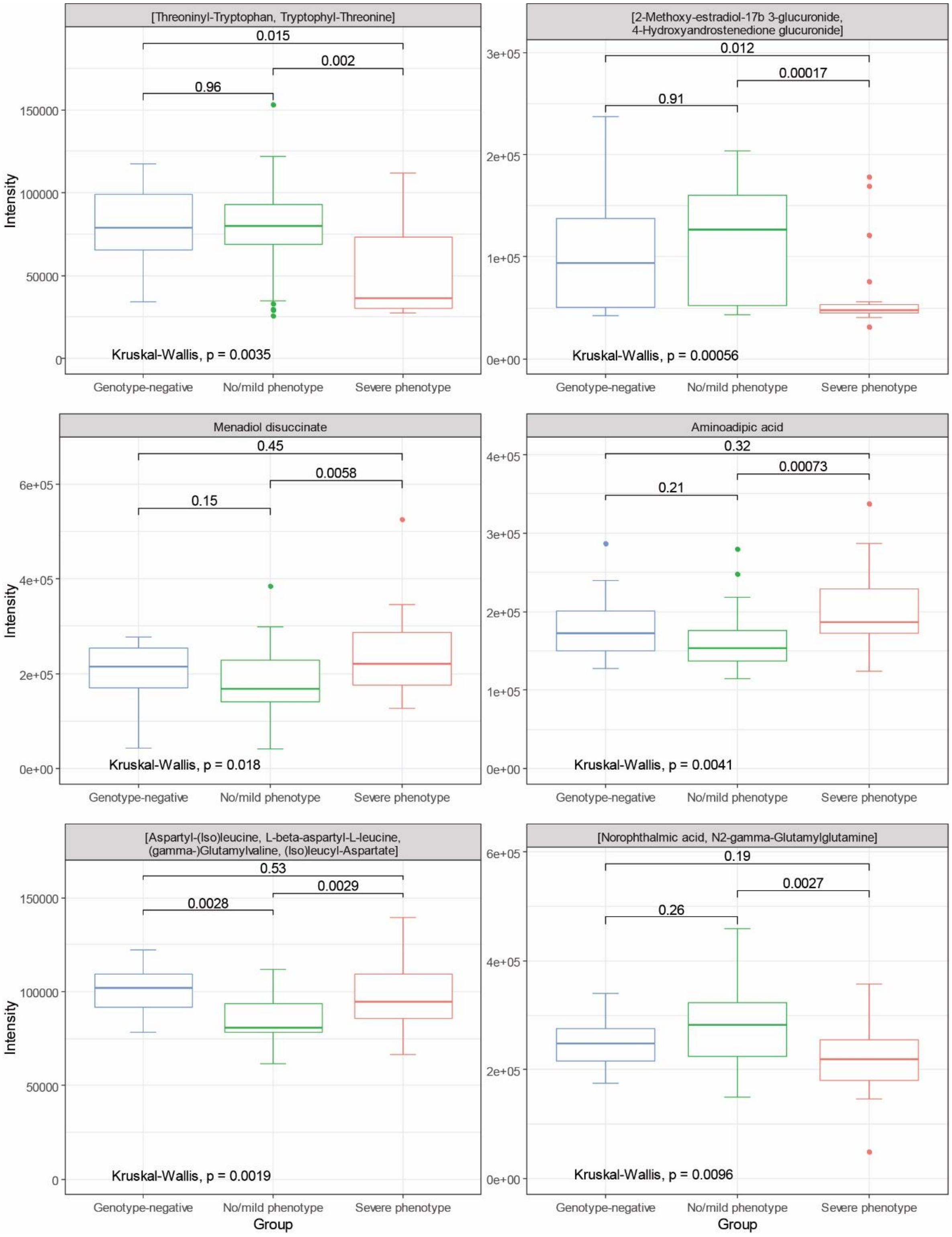

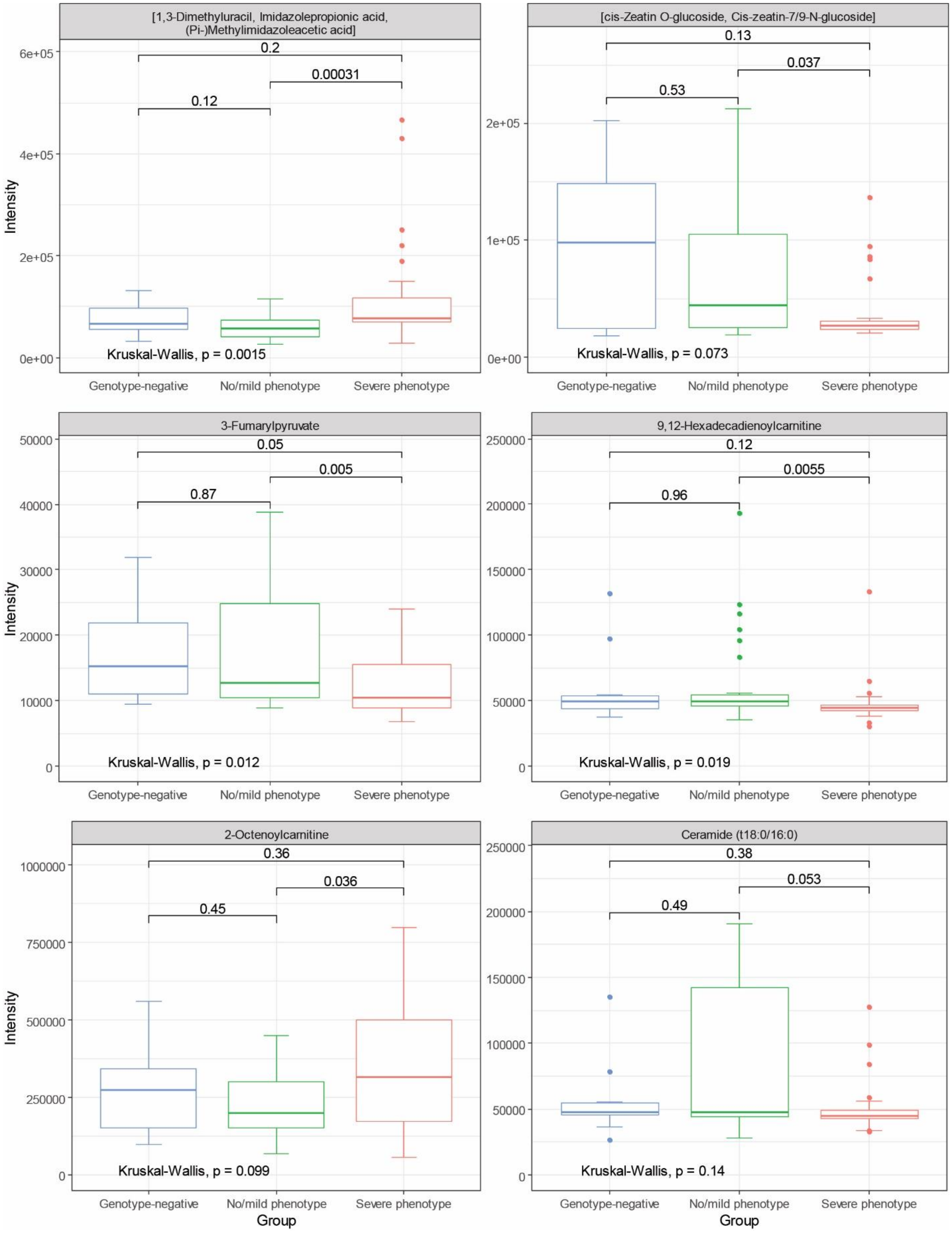
[1,3-Dimethyluracil, Imidazolepropionic acid, (Pi-)Methylimidazoleacetic acid]
0.2
0.00031
0.12
Kruskal-Wallis, p = 0.0015
[cis-Zeatin O-glucoside, Cis-zeatin-7/9-N-glucoside]
0.13
0.037
0.53
Kruskal-Wallis, p = 0.073
3-Fumarylpyruvate
0.05
0.005
0.87
Kruskal-Wallis, p = 0.012
9,12-Hexadecadienoylcarnitine
0.12
0.0055
0.96
Kruskal-Wallis, p = 0.019
2-Octenoylcarnitine
0.36
0.036
0.45
Kruskal-Wallis, p = 0.099
Ceramide (t18:0/16:0)
0.38
0.053
0.49
Kruskal-Wallis, p = 0.14
Intensity
Genotype-negative
No/mild phenotype
Severe phenotype
Group

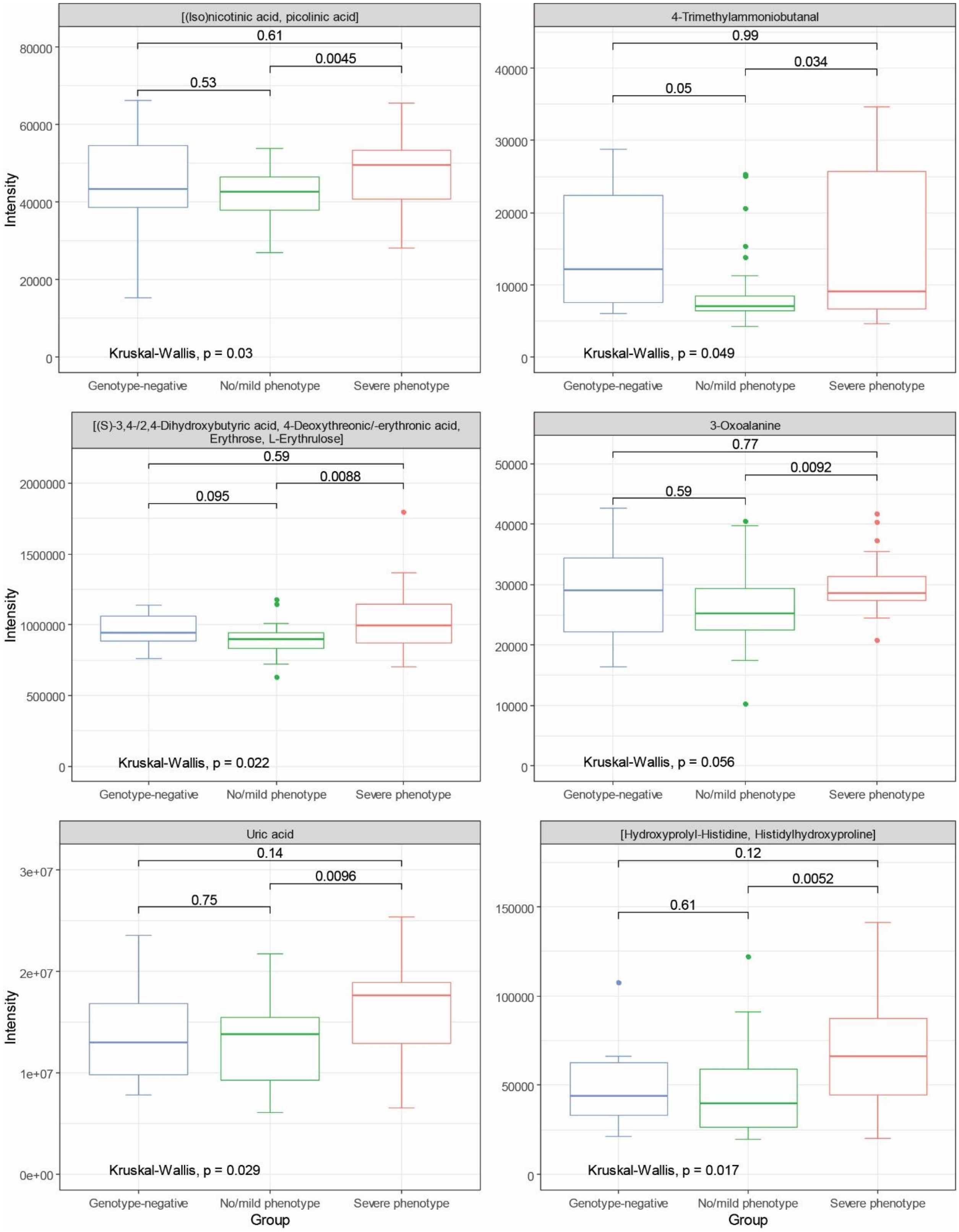

[(Iso)nicotinic acid, picolinic acid]
0.61
0.0045
0.53
Kruskal-Wallis, p = 0.03
4-Trimethylammoniobutanal
0.99
0.034
0.05
Kruskal-Wallis, p = 0.049
[(S)-3,4-/2,4-Dihydroxybutyric acid, 4-Deoxythreonic/-erythronic acid, Erythrose, L-Erythrulose]
0.59
0.0088
0.095
Kruskal-Wallis, p = 0.022
3-Oxoalanine
0.77
0.0092
0.59
Kruskal-Wallis, p = 0.056
Uric acid
0.14
0.0096
0.75
Kruskal-Wallis, p = 0.029
[Hydroxyprolyl-Histidine, Histidylhydroxyproline]
0.12
0.0052
0.61
Kruskal-Wallis, p = 0.017
Intensity
Genotype-negative
No/mild phenotype
Severe phenotype
Group

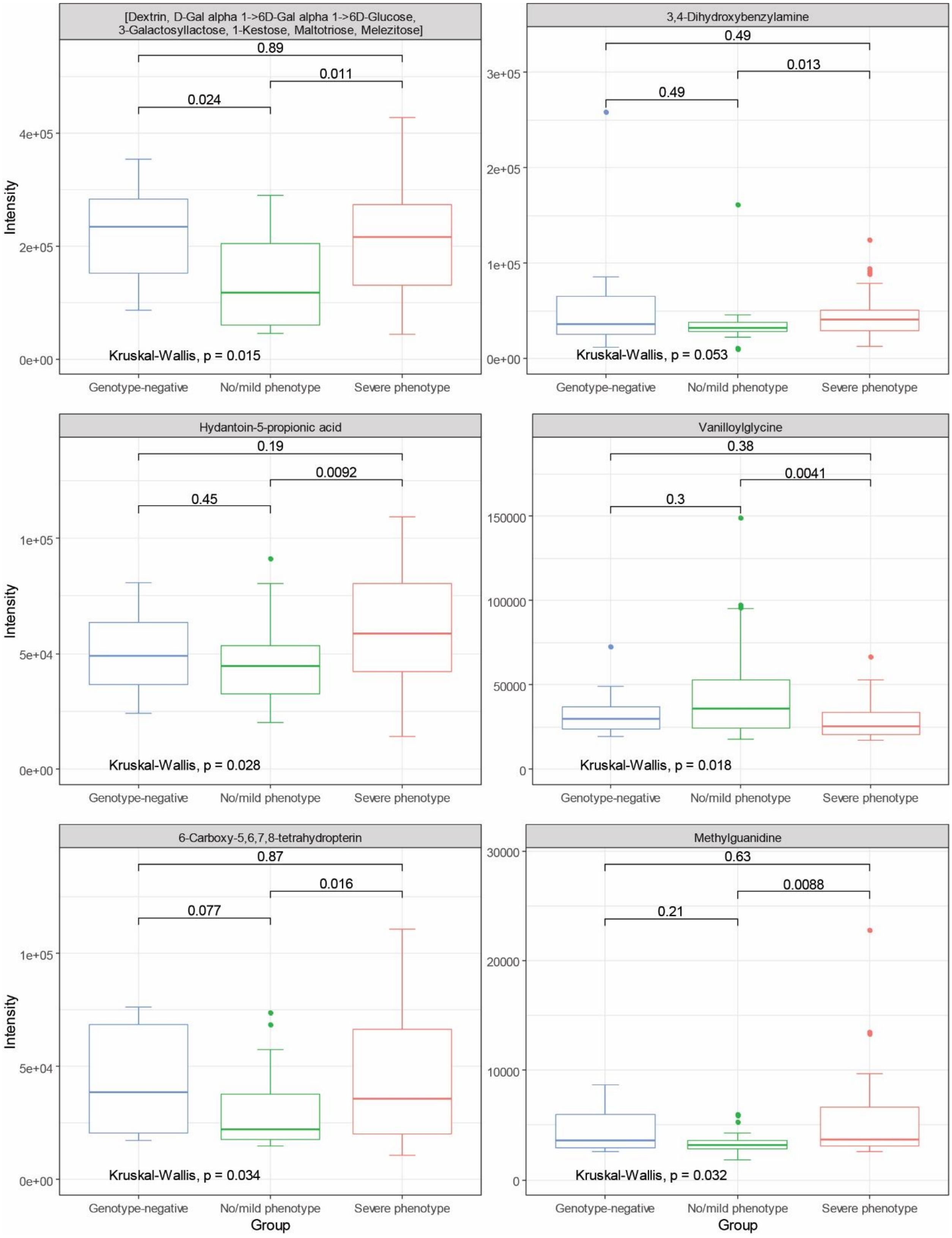

[Dextrin, D-Gal alpha 1->6D-Gal alpha 1->6D-Glucose, 3-Galactosyllactose, 1-Kestose, Maltotriose, Melezitose]
0.89
0.011
0.024
Kruskal-Wallis, p = 0.015
3,4-Dihydroxybenzylamine
0.49
0.013
0.49
Kruskal-Wallis, p = 0.053
Hydantoin-5-propionic acid
0.19
0.0092
0.45
Kruskal-Wallis, p = 0.028
Vanilloylglycine
0.38
0.0041
0.3
Kruskal-Wallis, p = 0.018
6-Carboxy-5,6,7,8-tetrahydropterin
0.87
0.016
0.077
Kruskal-Wallis, p = 0.034
Methylguanidine
0.63
0.0088
0.21
Kruskal-Wallis, p = 0.032
Intensity
Genotype-negative
No/mild phenotype
Severe phenotype
Group

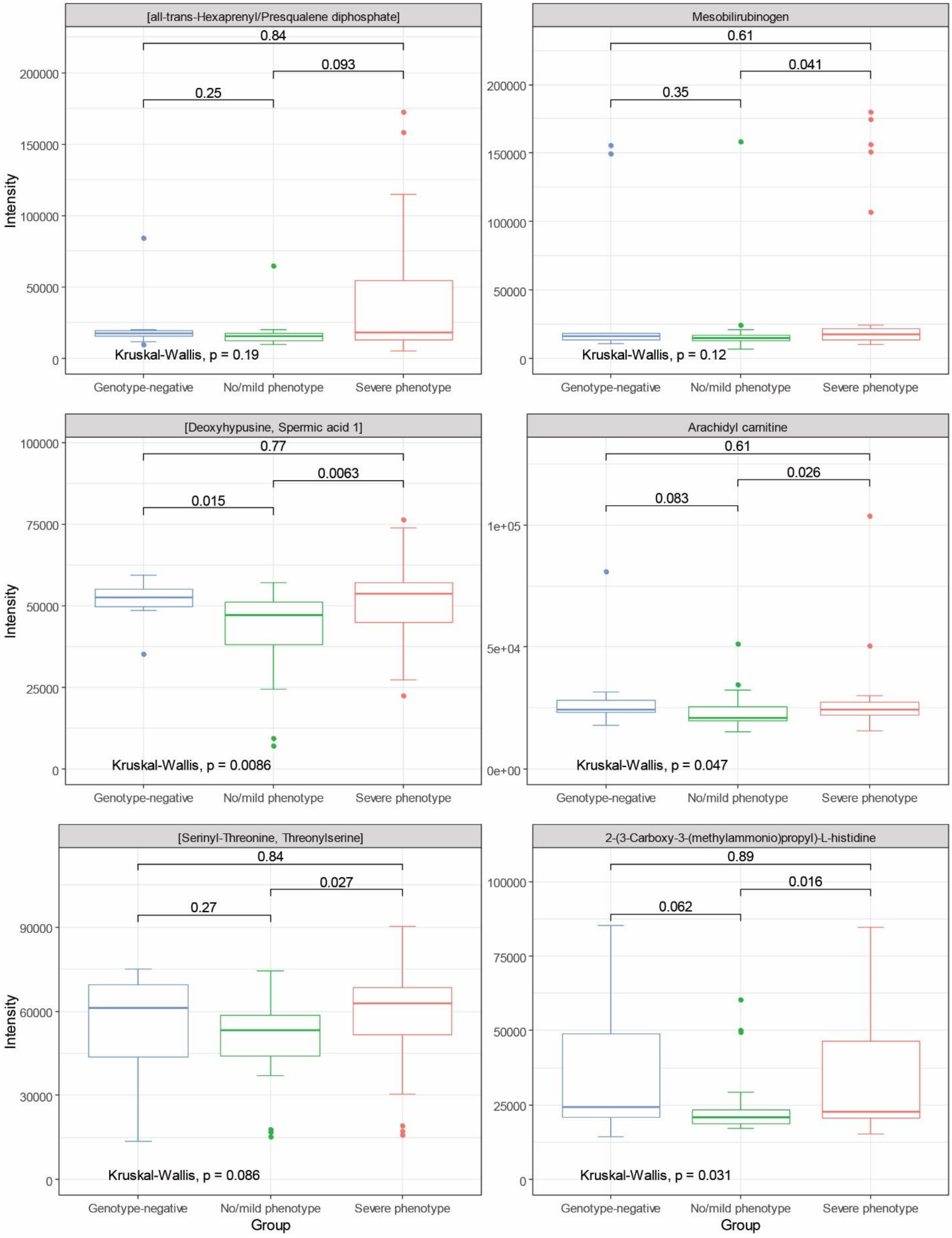
[all-trans-Hexaprenyl/Presqualene diphosphate]
0.84
0.093
0.25
Kruskal-Wallis, p = 0.19
Mesobilirubinogen
0.61
0.041
0.35
Kruskal-Wallis, p = 0.12
[Deoxyhypusine, Spermic acid 1]
0.77
0.0063
0.015
Kruskal-Wallis, p = 0.0086
Arachidyl carnitine
0.61
0.026
0.083
Kruskal-Wallis, p = 0.047
[Serinyl-Threonine, Threonylserine]
0.84
0.027
0.27
Kruskal-Wallis, p = 0.086
2-(3-Carboxy-3-(methylammonio)propyl)-L-histidine
0.89
0.016
0.062
Kruskal-Wallis, p = 0.031
Intensity
Genotype-negative
No/mild phenotype
Severe phenotype
Group

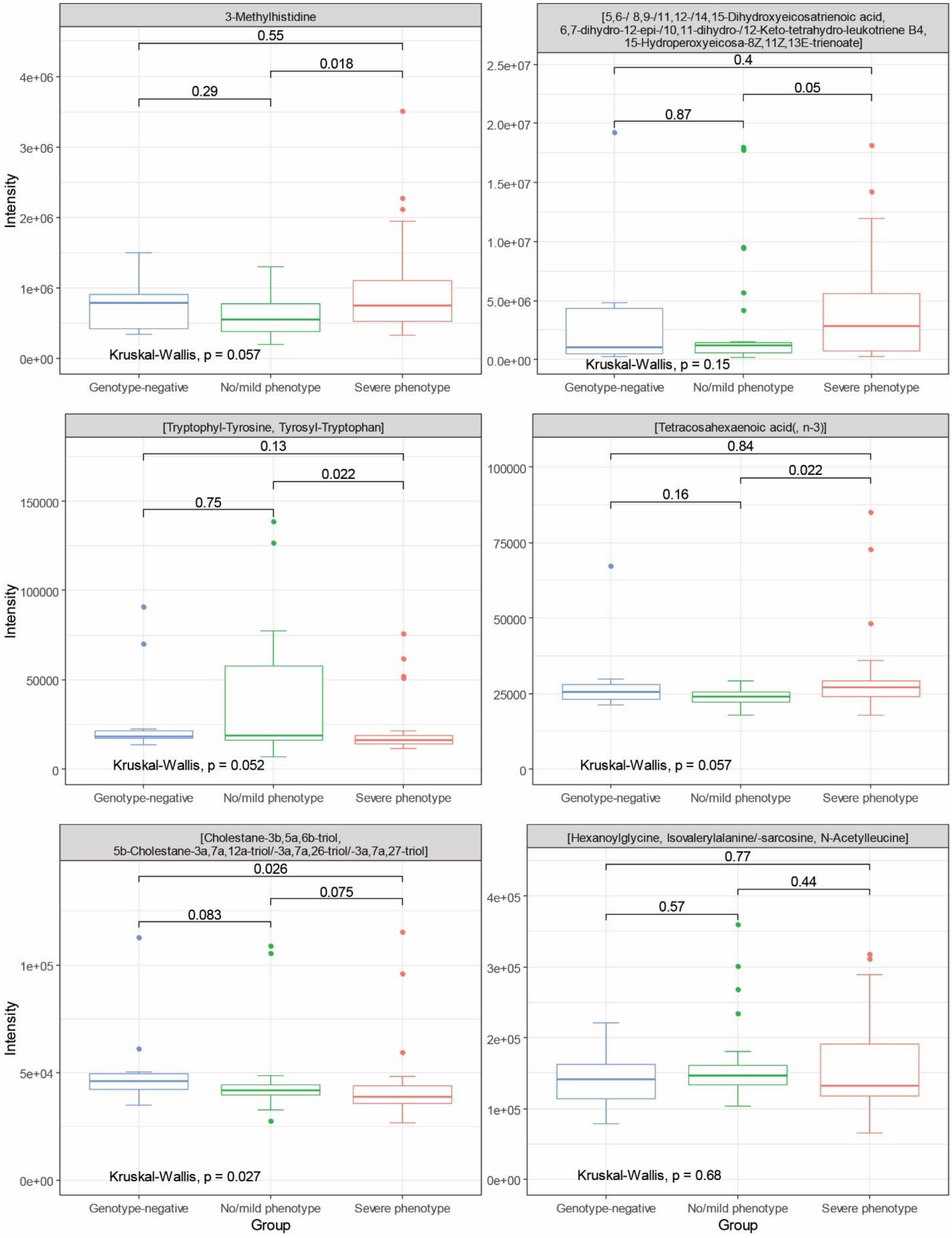
3-Methylhistidine
0.55
0.018
0.29
Kruskal-Wallis, p = 0.057
[5,6-/ 8,9-/11,12-/14,15-Dihydroxyeicosatrienoic acid, 6,7-dihydro-12-epi-/10,11-dihydro-/12-Keto-tetrahydro-leukotriene B4, 15-Hydroperoxyeicosa-8Z,11Z,13E-trienoate]
0.4
0.05
0.87
Kruskal-Wallis, p = 0.15
[Tryptophyl-Tyrosine, Tyrosyl-Tryptophan]
0.13
0.022
0.75
Kruskal-Wallis, p = 0.052
[Tetracosahexaenoic acid(, n-3)]
0.84
0.022
0.16
Kruskal-Wallis, p = 0.057
[Cholestane-3b,5a,6b-triol, 5b-Cholestane-3a,7a,12a-triol/-3a,7a,26-triol/-3a,7a,27-triol]
0.026
0.075
0.083
Kruskal-Wallis, p = 0.027
[Hexanoylglycine, Isovalerylalanine/-sarcosine, N-Acetylleucine]
0.77
0.44
0.57
Kruskal-Wallis, p = 0.68
Intensity
Genotype-negative
No/mild phenotype
Severe phenotype
Group

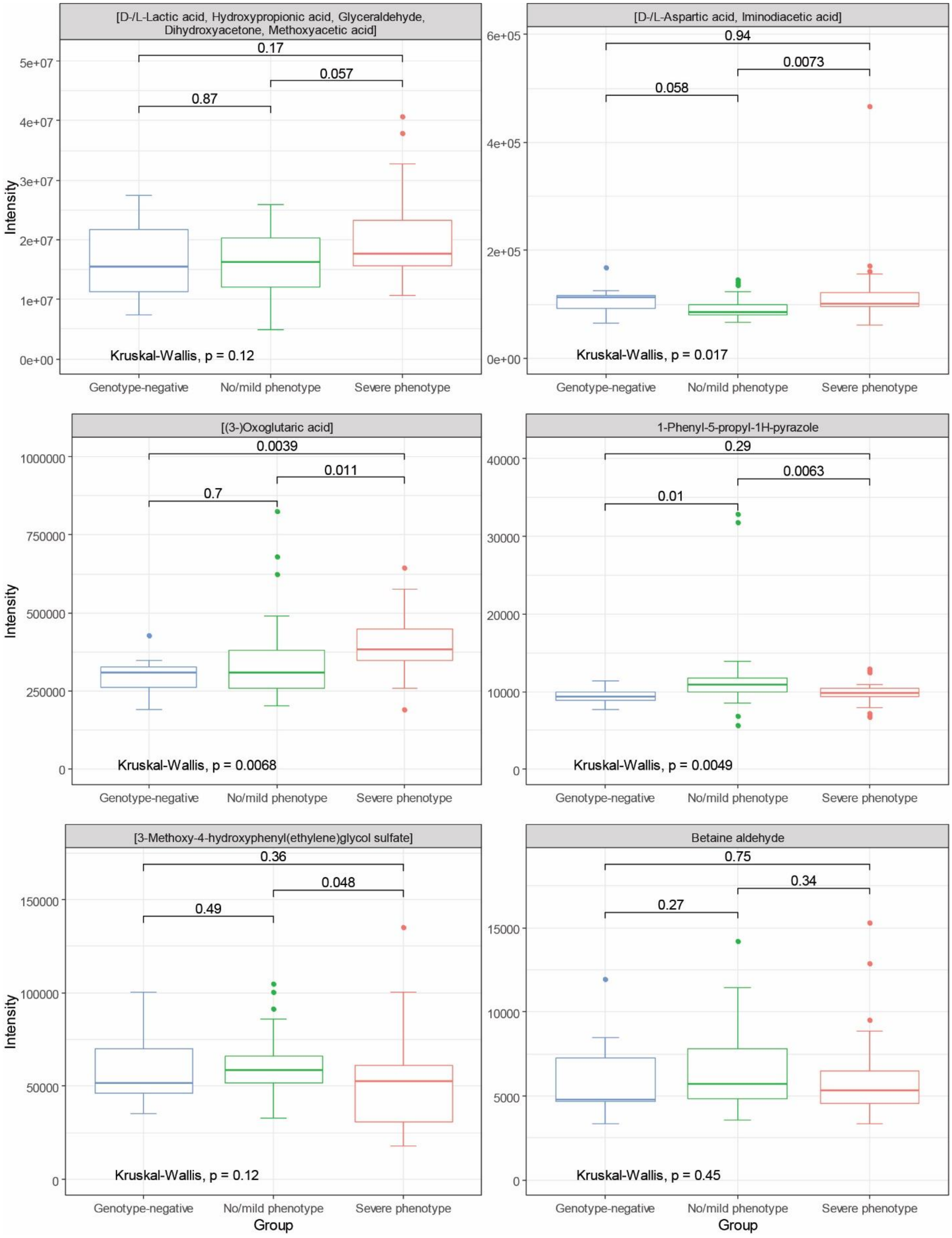
[D-/L-Lactic acid, Hydroxypropionic acid, Glyceraldehyde, Dihydroxyacetone, Methoxyacetic acid]
0.17
0.057
0.87
Kruskal-Wallis, p = 0.12
[D-/L-Aspartic acid, Iminodiacetic acid]
0.94
0.0073
0.058
Kruskal-Wallis, p = 0.017
[(3-)Oxoglutaric acid]
0.0039
0.011
0.7
Kruskal-Wallis, p = 0.0068
1-Phenyl-5-propyl-1H-pyrazole
0.29
0.0063
0.01
Kruskal-Wallis, p = 0.0049
[3-Methoxy-4-hydroxyphenyl(ethylene)glycol sulfate]
0.36
0.048
0.49
Kruskal-Wallis, p = 0.12
Betaine aldehyde
0.75
0.34
0.27
Kruskal-Wallis, p = 0.45
Intensity
Genotype-negative
No/mild phenotype
Severe phenotype
Group

Box plots of the intensities of the 42 top peaks identified by the biomarker identification models (sparse partial least squares discriminant analysis, XGBoost and Lasso logistic regression), stratified per group and ordered by relative importance across the three models. P-values are annotated for between group comparisons using Mann-Whitney U tests and across group comparisons using Kruskal-Wallis test.

Figure S5. Box plots of selected peaks, excluding subjects with heart failure

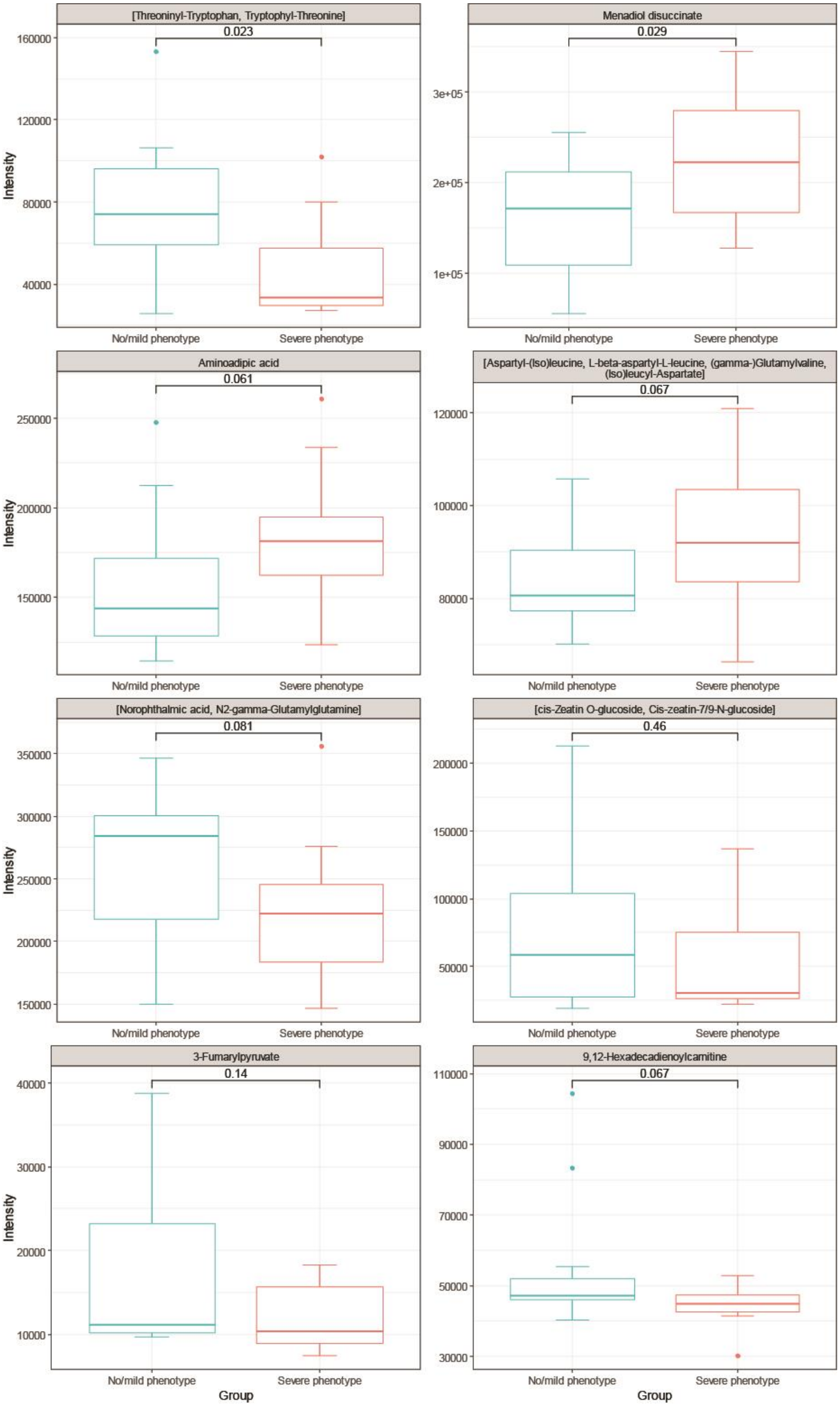

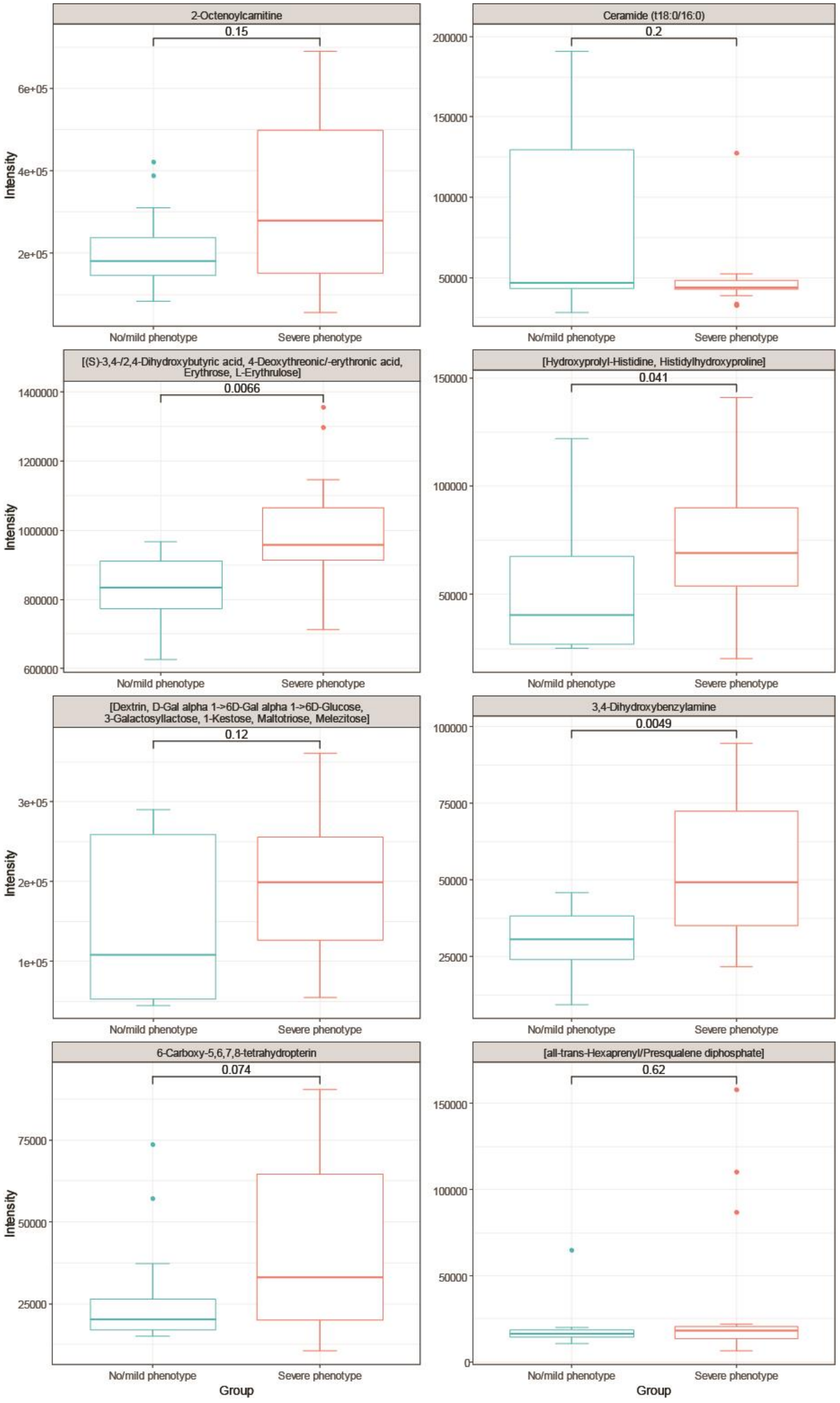
2-Octenoylcarnitine
0.15
Intensity
6e+05
4e+05
2e+05
No/mild phenotype
Severe phenotype
Ceramide (t18:0/16:0)
0.2
200000
150000
100000
50000
No/mild phenotype
Severe phenotype
[(S)-3,4-/2,4-Dihydroxybutyric acid, 4-Deoxythreonic/-erythronic acid, Erythrose, L-Erythrulose]
0.0066
Intensity
1400000
1200000
1000000
800000
600000
No/mild phenotype
Severe phenotype
[Hydroxyprolyl-Histidine, Histidylhydroxyproline]
0.041
150000
100000
50000
No/mild phenotype
Severe phenotype
[Dextrin, D-Gal alpha 1->6D-Gal alpha 1->6D-Glucose, 3-Galactosyllactose, 1-Kestose, Maltotriose, Melezitose]
0.12
Intensity
3e+05
2e+05
1e+05
No/mild phenotype
Severe phenotype
3,4-Dihydroxybenzylamine
0.0049
100000
75000
50000
25000
No/mild phenotype
Severe phenotype
6-Carboxy-5,6,7,8-tetrahydropterin
0.074
Intensity
75000
50000
25000
No/mild phenotype
Severe phenotype
Group
[all-trans-Hexaprenyl/Presqualene diphosphate]
0.62
150000
100000
50000
0
No/mild phenotype
Severe phenotype
Group

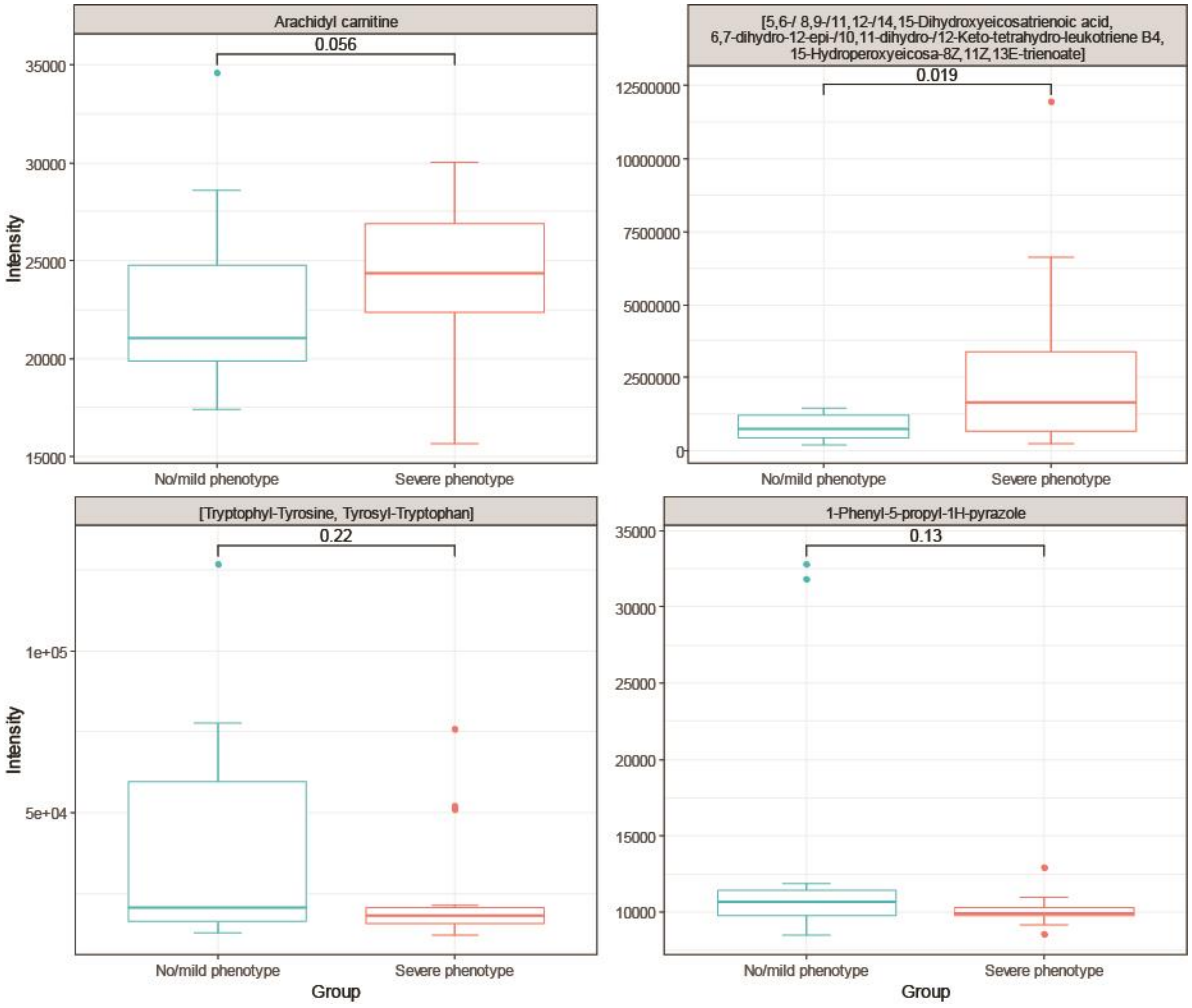

Box plots of the metabolites selected using Lasso logistic regression in the sensitivity analysis excluding subjects with heart failure. P-values are annotated for between group comparisons using Mann-Whitney U tests.

Figure S6. Box plots of selected peaks, excluding subjects with prior septal reduction therapy

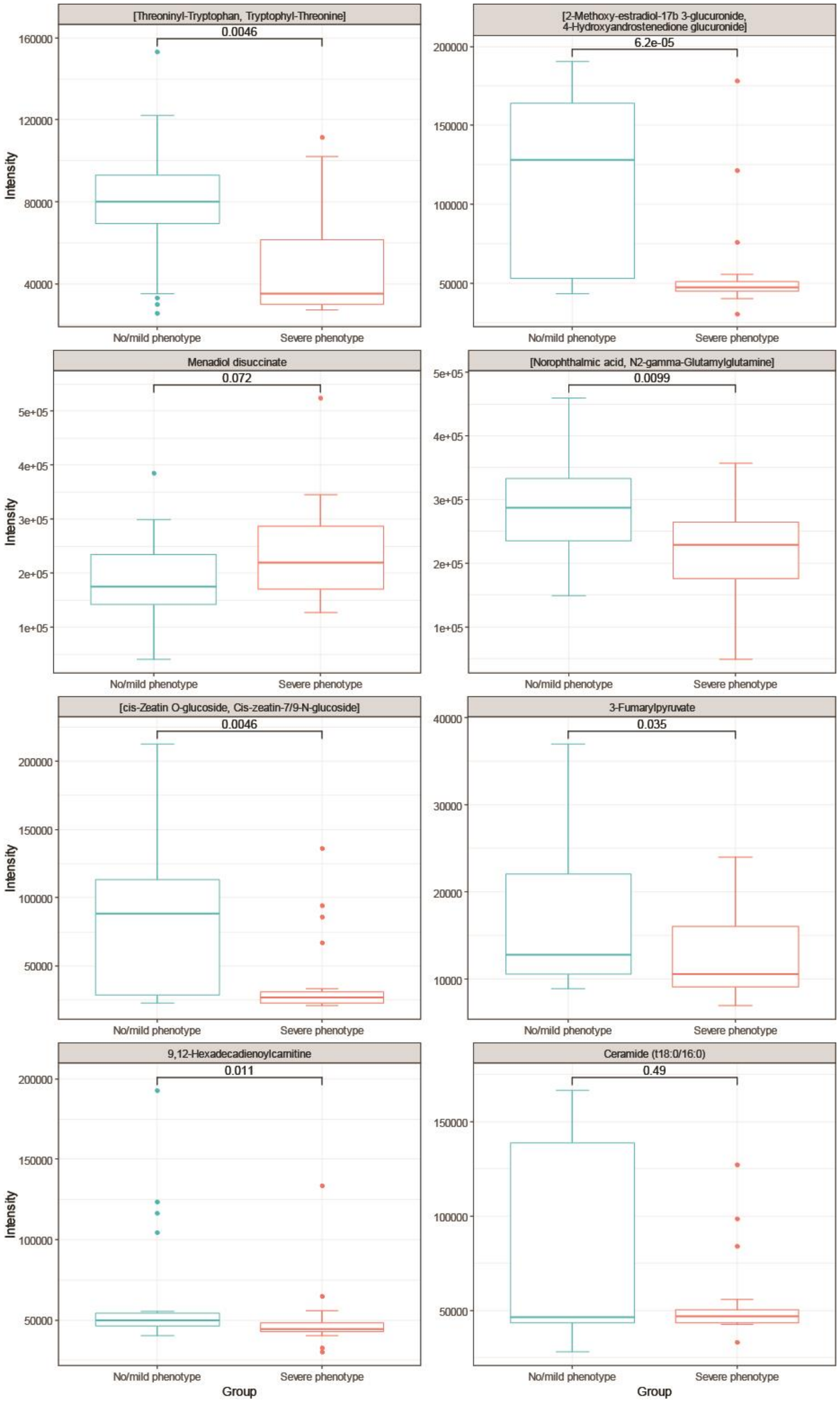

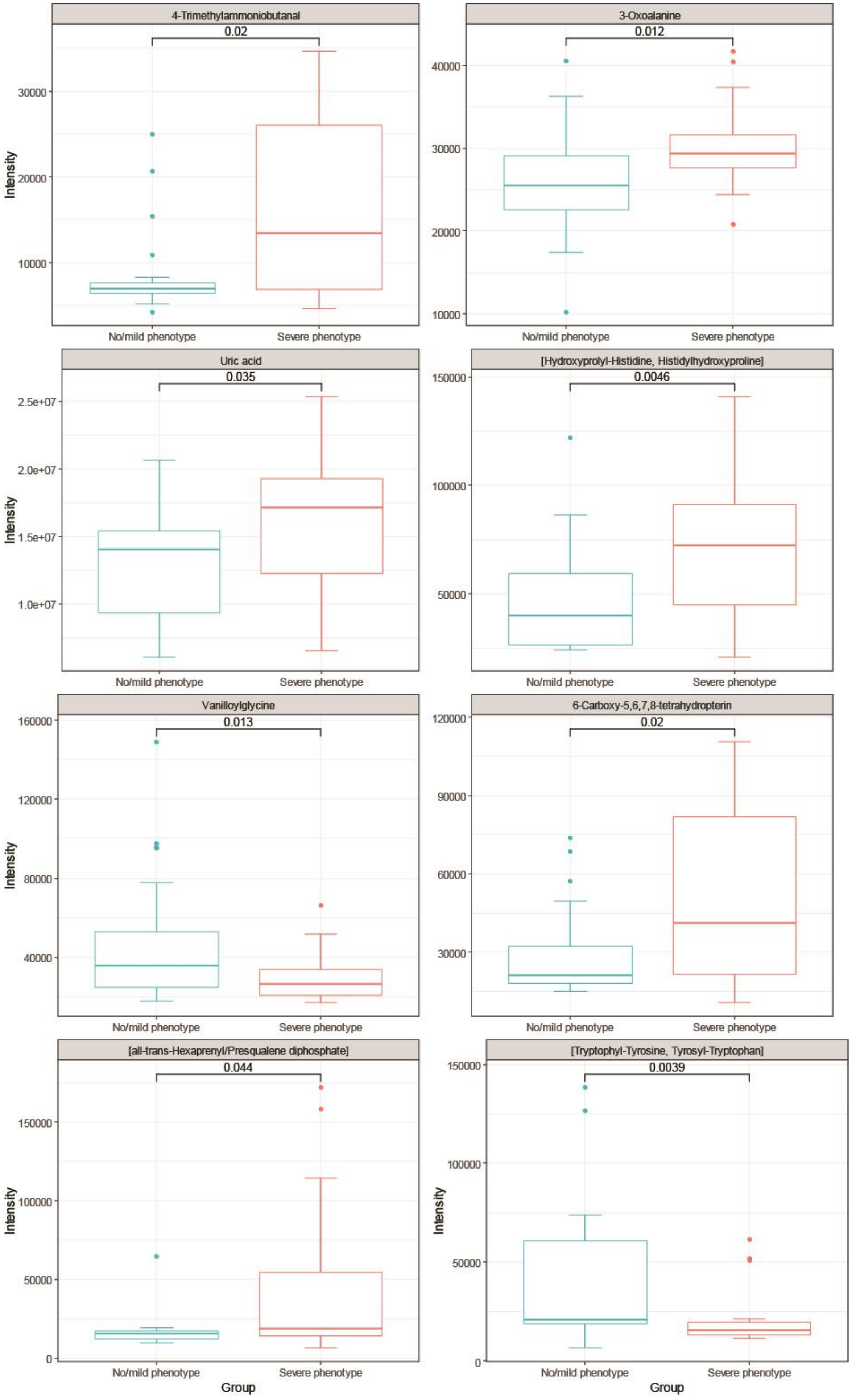
4-Trimethylammoniobutanal
0.02
3-Oxoalanine
0.012
Uric acid
0.035
[Hydroxyprolyl-Histidine, Histidylhydroxyproline]
0.0046
Vanilloylglycine
0.013
6-Carboxy-5,6,7,8-tetrahydropterin
0.02
[all-trans-Hexaprenyl/Presqualene diphosphate]
0.044
[Tryptophyl-Tyrosine, Tyrosyl-Tryptophan]
0.0039
Intensity
No/mild phenotype
Severe phenotype
Group

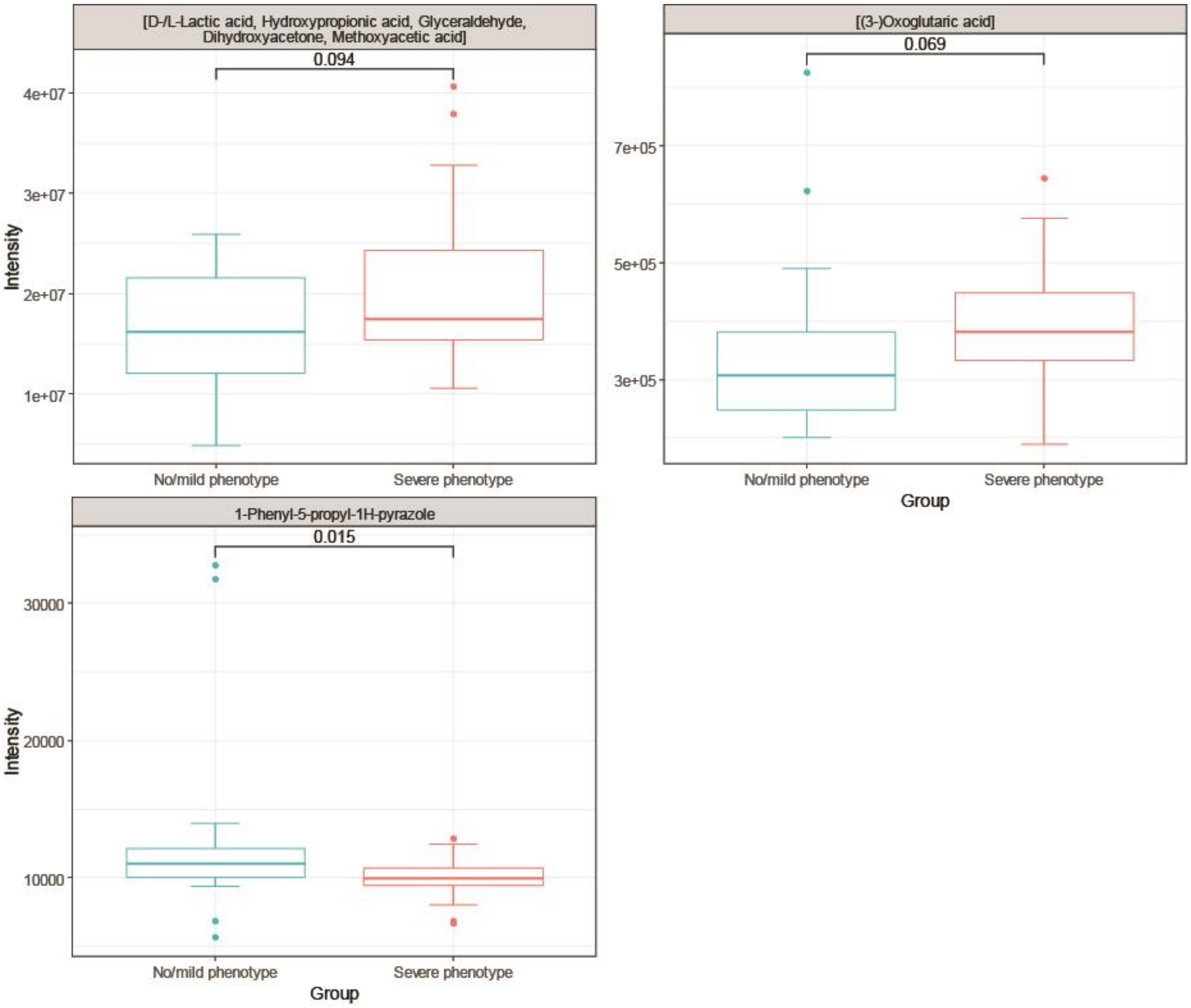

Box plots of the metabolites selected using Lasso logistic regression in the sensitivity analysis excluding subjects with prior septal reduction therapy. P-values are annotated for between group comparisons using Mann-Whitney U tests.
